# Comment on “A novel super-enhancer-related gene signature predicts prognosis and immune microenvironment for breast cancer”

**DOI:** 10.1186/s12885-024-12124-w

**Published:** 2024-03-25

**Authors:** Matin Chehelgerdi, Milad Khorramian-Ghahfarokhi, Fereshteh Behdarvand Dehkordi, Mohammad Chehelgerdi

**Affiliations:** 1 Novin Genome (NG) Lab, Research and Development Center for Biotechnology, Shahrekord, Iran; 2 Young Researchers and Elite Club, Shahrekord Branch, Islamic Azad University, Shahrekord, Iran; 3 Division of Biotechnology, Department of Pathobiology, School of Veterinary Medicine, Shiraz University, Shiraz, Iran

**Keywords:** Breast cancer, Prognosis, Super-enhancer, Gene signature, Immune microenvironment, RNA-sequencing, Heterogeneity, Molecular subtypes, Benchmarking, Immunotherapy, Triple-negative breast cancer

## Abstract

The primary aim of this study is to critically evaluate and comment on the research presented in the article titled “A Novel Super-Enhancer-Related Gene Signature Predicts Prognosis and Immune Microenvironment for Breast Cancer” by Wu et al. Our specific objectives include assessing the methodology employed by the authors, particularly in regard to the utilization of a super-enhancer-related gene signature for breast cancer prognosis prediction. We propose the necessity of subgroup analysis to effectively address the heterogeneity in breast cancer subtypes, which is crucial for the applicability of the SERGs across diverse breast cancer cases. Additionally, we suggest conducting a more comprehensive immune panel study to deepen the understanding of how the immune microenvironment impacts breast cancer prognosis. Our commentary seeks to provide valuable insights into the strengths and weaknesses of the study, contributing to a more comprehensive understanding of its findings and potential clinical implications.

## Dear editor

I would like to offer a comparative analysis of the recently published study titled “A Novel Super-Enhancer-Related Gene Signature Predicts Prognosis and Immune Microenvironment for Breast Cancer” by Qing Wu, Xuan Tao, Yang Luo, Shiyao Zheng, Nan Lin, and Xianhe Xie (BMC Cancer volume 23, Article number: 776, 2023). In light of the study’s findings, it is essential to consider its contributions alongside related research to provide a comprehensive perspective on the topic.

Wu et al. [1] presents a unique approach to prognosis prediction by utilizing a super-enhancer-related gene signature (SERGs) in the context of breast cancer. The authors establish a prognostic signature based on six genes: ZIC2, NFE2, FOXJ1, KLF15, POU3F2, and SPIB. While this is a pioneering effort, there are notable comparisons and insights to be drawn from previous research.

Firstly, the Wu et al. utilized RNA-sequencing data from The Cancer Genome Atlas (TCGA) and established a prognostic signature using six SERGs. While the approach is valid, it is essential to acknowledge the potential heterogeneity within the TCGA dataset itself. To this end, we emphasize the necessity of conducting subgroup analyses. Such analyses would provide more accurate insights into the signature’s effectiveness across different breast cancer subtypes, potentially revealing varied prognostic implications based on these subtypes. Breast cancer is a highly heterogeneous disease with various molecular subtypes that can impact prognosis. Without proper subtype-specific analysis, the applicability of the proposed signature to diverse breast cancer subtypes may be limited.

Comparatively, it is worth noting that other prognostic gene expression signatures have been developed and compared in the context of breast cancer prognosis. However, to present something novel or more persuasive, it is crucial to not only benchmark the SERGs signature against these established signatures but also to integrate a more comprehensive immune panel study. This would enable a deeper exploration into how the immune microenvironment affects breast cancer prognosis, potentially leading to more impactful conclusions. In particular, the work by Haibe-Kains et al. [2] compared the prognostic performance of three gene expression signatures, revealing agreement and overlapping prediction rates. This comparative aspect highlights the importance of benchmarking the proposed SERGs signature against other established signatures on independent patient cohorts, shedding light on its relative predictive power. Furthermore, the authors emphasize the immune microenvironment prediction capacity of their SERGs signature. However, direct comparison with existing immune-related gene signatures could offer a more comprehensive understanding of its strengths and limitations. For example, the study by Liu et al. [3] developed an immune checkpoint-related gene signature specifically for triple-negative breast cancer (TNBC), showcasing both prognostic and immune status prediction capabilities. Considering the evolving landscape of immunotherapy, such comparisons would provide valuable insights into the unique contributions of the SERGs signature in the context of immune-related predictions.

Breast cancer is categorized into various types and subtypes, determined by characteristics such as specific receptors, gene expression patterns, and histological features. The focus is solely on patients with Her2 + breast cancer, without specifying the different groups involved [4]. 

ZIC2, a gene, shows notably higher expression in the BT549 cell line compared to the MCF7 cell line. The BT549 cell line belongs to the basal-like subtype, while MCF7 represents the luminal A subtype. These subtypes are characterized by unique molecular attributes and distinct gene expression patterns. The variation in ZIC2 expression between these subtypes may stem from differences in their regulatory mechanisms or the signaling pathways unique to each subtype. Genetic variations commonly found in breast cancer cell lines, such as mutations, amplifications, deletions, or epigenetic changes, could influence the expression of ZIC2. These genetic differences between BT549 and MCF7 may affect the regulation of ZIC2 expression, as observed by Makki 2015, leading to different expression levels of the ZIC2 gene in the two cell lines.

Breast cancer, being a multifaceted and diverse illness, consists of various subtypes and exhibits a wide range of molecular characteristics. The super-enhancers and their corresponding genes can vary significantly among these subtypes and from patient to patient. This variability poses a significant challenge in pinpointing consistent and dependable biomarkers [5]. The relationship between SERS and tumor characteristics such as Tumor Mutational Burden (TMB), mutation counts, and copy number burdens remains unexplored in both groups. In breast cancer cases with low TMB, fewer somatic mutations are typically found in the coding regions of the tumor genome. Generally, breast cancer has a lower TMB compared to other cancer types. However, in breast cancer treatment, TMB might not be the key biomarker for selecting targeted therapies, as other genetic alterations and biomarkers like hormone receptor or HER2 status could be more significant. TMB has become notable for its role in predicting the response to immunotherapy, especially immune checkpoint inhibitors. It’s observed that tumors with higher TMB are more likely to produce neoantigens, which are new antigens capable of triggering a more robust immune response. This can make immunotherapies more effective [6, 7].

Super enhancers, heavily reliant on specific transcription co-factors like BET and BRD4 in each cell and tissue, play a crucial role in defining and maintaining cell and tissue identity. These super enhancers, especially those containing cell-type-specific master transcription factors, are often associated with genes that determine cell identity [8]. They are vital in managing mammalian cell identity but can also change dynamically in response to various stimuli, treatments, or during disease progression. The persistence and uniformity of ZIC2-associated super enhancers across different times and conditions is a subject of research, important for confirming ZIC2’s reliability as a prognostic biomarker [9]. However, the lack of detailed information about patients’ treatment histories, including radiotherapy or drug treatments, limits the effectiveness of ZIC2 as a prognosis predictor. Moreover, super enhancers and their related genes can have varying effects depending on the context. The significance of ZIC2, as a gene linked to super enhancers, may differ based on cellular circumstances, genetic makeup, and environmental factors. Hence, it’s essential to understand these context-dependent impacts to accurately assess ZIC2’s prognostic value [10]. In the environment of a tumor, the battle for nutrients between immune cells and cancer cells plays a key role in determining the tumor’s outcome. It’s important to link Surface Enhanced Raman Scattering (SERS) with changes in metabolic pathways or network alterations. Such metabolic exchanges can influence how immune cells operate, the advancement of the tumor, and the effectiveness of treatments [11].

In conclusion, while the study by Wu et al. presents an innovative approach, we highlight the necessity of addressing potential dataset heterogeneity through subgroup analysis and the importance of conducting a more comprehensive immune panel study to enhance the understanding of the immune microenvironment’s role in breast cancer. However, to ensure its clinical applicability and robustness, it is crucial to address potential heterogeneity within the dataset, benchmark its performance against other prognostic signatures, and compare its immune-related prediction capacity with established immune gene signatures. Such considerations would contribute to a more holistic interpretation of the study’s findings and their potential implications.

## Data Availability

Not applicable.
